# Hyperchloremic metabolic acidosis caused by a large influx of saline into the peritoneal and retroperitoneal cavities during transurethral resection

**DOI:** 10.1186/s40981-022-00580-x

**Published:** 2022-11-22

**Authors:** Mariko Muto, Chiaki Nemoto, Satoki Inoue

**Affiliations:** 1 Department of Anesthesiology, Ohara General Hospital, 6-1 Uwamachi, Fukushima, 960-8611 Japan; 2 Department of Anesthesiology, Fukushima Medical University School of Medicine, 1 Hikarigaoka, Fukushima, 960-1295 Japan

To the Editor,

Transurethral resection in saline (TURis) systems tends to be adopted to avoid transurethral resection syndrome due to the systemic absorption of nonelectrolyte perfusate [1]. Nonetheless, hyperchloremic metabolic acidosis (HCA) due to rapid intravascular absorption of perfusate has also been reported with TURis [2]. However, there are no reports showing HCA due to the slow absorption of intraperitoneal saline. We report a case of HCA due to an intraperitoneal influx of perfused saline during TURis.

A 90-year-old woman (40.8 kg; 142.6 cm) was scheduled for transurethral en bloc resection of a superficial bladder tumor because of multiple tumors extending from the bladder triangle to the posterior bladder wall [3]. General anesthesia was induced and maintained with remimazolam, remifentanil, and rocuronium. Ventilation was performed using a volume-controlled mode (6–8 ml/kg). During the surgery, the patient's blood pressure decreased, and continuous phenylephrine was administered. Twenty minutes after the start of the surgery, her end-tidal carbon dioxide (ETCO_2_) concentration rose, and her airway pressure increased, so the ventilatory mode was adjusted. A total of 16 L of saline was used for bladder perfusion, and 810 ml of crystalloids was infused. The recovery amount of bladder perfusate was not calculated. The operating time was 91 min. Postoperatively, the patient was awakened and extubated, but required resedation and reintubation due to abdominal distension and restlessness. Abdominal ultrasonography revealed the presence of a large amount of fluid around the kidney and liver, suggesting symptoms due to the influx of saline into the peritoneal and retroperitoneal cavities during bladder irrigation. The drainage of ascites was difficult, so we decided to wait for a spontaneous resolution. After reintubation, a continuous administration of noradrenaline was performed because the patient’s hypotension persisted. Blood gas analysis after reintubation revealed HCA (Table 1). The patient's respiratory and hemodynamic status gradually stabilized, and her postoperative urine output was 7300 ml in 38 h, with an infusion volume of 800 ml. Sixteen hours after surgery, blood gas analysis showed that metabolic acidosis continued, but it was compensated by respiratory alkalosis. The patient did not complain of abdominal distention, and her respiratory status was stable after extubation. The patient was discharged from the hospital on postoperative day 19.

**Table 1:** Blood collection date

	Preoperative	At ICU admission (FiO_2_: 0.35)	On the first postoperative day (FiO_2_: 0.35)
Hb (g/dL)	8.4	8.4	8.4
TP (g/dL)	7.3	n/a	6.5
Na (mmol/L)	141	143	142
K (mmol/L)	4.6	3.6	4.3
Cl (mmol/L)	106	117	115
pH	n/a	7.185	7.341
HCO_3-_ (mmol/L)	n/a	15.4	18.7
Lactate (mg/dl)	n/a	27	15
Actual base excess effect (mmol/L)	n/a	-12	-6.4
PaO_2_ (mmHg)	n/a	74.9	111
PaCO_2_ (mmHg)	n/a	40.8	34.6

This patient had fluid accumulation in the peritoneal and retroperitoneal cavities after TURis, and blood gas analysis after reintubation showed HCA. A case of dilute HCA with the rapid transvascular absorption of perfusion saline during TURis has been reported [2]. In this case, it is thought that HCA was caused by the large volume of saline in the peritoneal and retroperitoneal cavities. Acidosis may be associated with the development of restlessness and hypotension, as reported by Okuma et al. [2] In addition, since the patient’s symptoms may also have been due to increased intra-abdominal pressure, monitoring abdominal pressure with measured bladder pressure may have been useful for pathology discrimination in this situation.

## Data Availability

Not applicable.

## Abbreviations

TURis: Transurethral resection in saline
HCA: Hyperchlemic metabolic acidosis
ETCO_2_: End-tidal carbon dioxide
